# Non-Alcoholic Fatty Liver Disease as a Predictor of Atrial Fibrillation in Middle-Aged Population (OPERA Study)

**DOI:** 10.1371/journal.pone.0142937

**Published:** 2015-11-16

**Authors:** Aki J. Käräjämäki, Olli-Pekka Pätsi, Markku Savolainen, Y. Antero Kesäniemi, Heikki Huikuri, Olavi Ukkola

**Affiliations:** Research Unit of Internal Medicine, Medical Research Center Oulu, Oulu University Hospital, and University of Oulu, Oulu, Finland; University of Verona, Ospedale Civile Maggiore, ITALY

## Abstract

Non-alcoholic fatty liver disease (NAFLD) and atrial fibrillation (AF) are widespread diseases and have multiple common risk factors and comorbidities. No studies of association between ultrasonography-diagnosed NAFLD and AF exist in other than diabetic population. The goal of this prospective study was to study the value of NAFLD as a predictor of atrial fibrillation. This study had 958 subjects from the OPERA (Oulu Project Elucidating Risk of Atherosclerosis) cohort, and the mean follow-up time was 16.3 years. NAFLD was diagnosed if the subject had fatty liver in ultrasonography and no excess alcohol intake. AF was followed in the National Registers. In this study 249 subjects (26.0%) had NAFLD and 37 (14.9%) of these had AF whereas only 56 (7.9%) of those without NAFLD experienced AF during the follow-up time (p = 0.001). In the multiple Cox regression analysis including potential confounders (age, sex, study group, diabetes, body mass index (BMI), waist circumference, alcohol consumption, smoking, serum alanine aminotransferase concentration (ALT), systolic blood pressure, quick index, left ventricular mass index, left atrial diameter, coronary artery disease (CAD), atrial natriuretic peptide (ANP) and high sensitive C-reactive protein (hs-CRP)), NAFLD remained as an independent predictor of AF (Adjusted OR, 1.88 (95% Confidence interval (CI) 1.03–3.45)). In conclusion, our data shows that NAFLD is independently associated with the risk of AF.

## Introduction

Fatty liver is defined as fat accumulation of at least 5% of liver weight, a condition called steatosis. In cases with no excess alcohol drinking, the steatosis is called non-alcoholic fatty liver disease (NAFLD). NAFLD is prevalent due to the universal epidemic of obesity. Approximately 20–30% of adult population in the Western countries has NAFLD, the prevalence increasing to up to 70–90% in those with obesity or diabetes [1]. NAFLD is the most common chronic liver disease in the general population, posing an enormous burden on public health-care costs [2, 3]. In recent years, a growing body of evidence has shown that NAFLD is also a systemic disease, being a remarkable risk factor of cardiovascular diseases, such as CAD, left ventricular (LV) diastolic dysfunction, excessive fat accumulation in the epicardial area, LV hypertrophy and aortic valve sclerosis [1, 4–6], as well as increased carotid-artery intimal medial thickness and prevalence of carotid atherosclerotic plaques [1]. Moreover, there is a strong association between NAFLD, metabolic syndrome (MS) and type 2 diabetes [7, 8]. Despite its high prevalence and clinical significance, knowledge and awareness of NAFLD is poor, not only among the general population but also among general practitioners [9].

AF is the most common cardiac arrhythmia, and due to an ageing population its prevalence is expected to rise in the future [10, 11]. The lifetime risk of AF is up to 25% and its prevalence nearly doubles with each decade of life [12]. There are many pathological conditions that are important risk factors for new-onset AF, such as obesity, hypertension, diabetes, CAD, valvular heart disease (VHD) and heart failure (HF) [13, 14]. The two most hazardous morbidities are stroke and heart failure [15], which are the reasons behind the decline of quality of life and high mortality rates. For instance, the age-adjusted relative risk of death attributable to AF is 1.6–1.87 world-wide [15–18], and the Euro Heart Survey (EHS) showed that in a one-year follow-up of over 5,300 adult-patients, all with AF, the all-cause death rate was as high as 5.3%, two-thirds of the deaths caused by a cardiovascular reason [19]. In addition to human suffering, the health-care costs of AF are considerable [16].

Recently, Targher et al. demonstrated in two different studies that NAFLD, diagnosed by ultrasonography, is associated with an increased prevalence and incidence of atrial fibrillation (AF) in patients with type 2 diabetes [20, 21]. Moreover, studies have shown that normal gamma-glutamyl transpeptidase (γ-GT) values are linearly associated with AF risk [22] and that high transaminases (ALT and Aspartate Aminotransferase (AST)) correlate with the risk of AF [23].

The aim of this prospective study, based on the OPERA material, was to find out whether subjects with NAFLD, diagnosed by ultrasonography, were at greater risk of AF than subjects without NAFLD. This study with 958 subjects was based on the OPERA population and the mean follow-up time was 16.3 years.

## Methods

### Study population

The study was approved by the Ethics Committee of the Medical Department of the University of Oulu (48/2009). Written informed consent was given by participants for their clinical records to be used in this study.

In the OPERA study, middle-aged hypertensive subjects and age- and sex-matched control subjects, none with medication for hypertension, were randomly selected (n = 1,045) from the national registries in the early 1990s [24]. The study was designed to evaluate the risk factors and occurrence of atherosclerotic cardiovascular diseases. There were 600 treated hypertensives (300 men and 300 women) who were randomly selected from the register of the Social Insurance Institute for the reimbursement of hypertension medication. All subjects were aged 40–59 years. Their age- and sex-matched controls, all living in Northern Finland in the city of Oulu and none of them with a verified need for medical treatment for hypertension, were also randomly selected from the registers of Social Insurance Institutes. However, subjects with non-diagnosed hypertension were observed in this middle-aged population. The subjects treated by non-pharmacological means for mild hypertension were accepted to the control group. All men were recruited between December 1990 and May 1992 and the women about one year later. Of these 1,200 subjects, 1,045 (520 men (261 from the hypertension group and 259 from the control group) and 525 women (258 from the hypertension group and 267 from the control group)) took part in the study. Thereby the overall participation rate was 87.1%. All study subjects visited the research laboratory of the Department of Internal Medicine, University of Oulu, where they underwent a clinical examination including height and weight measurements and a wide range of routine laboratory analyses were performed. At this first visit, a standardized health questionnaire covering the subjects’ past medical history, alcohol consumption, smoking, physical activity, current and former medication and family history was completed by two specially trained nurses and the details were checked by a physician later during the same visit. BMI was calculated as weight (kg) divided by height squared (m^2^). An automatic oscillometric blood pressure recorder (Dinamap, Critikon Ltd) was used to measure blood pressure from the right arm in a sitting position after an overnight fast and after 10 to 15 minutes’ rest. Three measurements were made at 1-minute intervals, and the means of the last two were used in the analyses. All subjects from the hypertensive groups were invited to the examinations without a prior pause of drug therapy. All these extensive examinations were performed during the years 1990–1993. CAD diagnosis was based on the data in the patients’ medical records.

Subjects with excessive alcohol intake (≥210g a week in men or ≥140g a week in women) according to criteria mentioned earlier [9] were not included in the present study. After this there were 969 subjects left, but ultrasonography data were missing for 11 subjects and they were excluded. Thereby 958 study subjects were enrolled in the study (472 subjects (49.3%) from the hypertensive group and 486 subjects (50.7%) from the control group; 450 men (47.0%) and 508 women (53%)). The mean age of the patients was 51.3 years, (range 40.2–62.0 years).

Moreover, in this study we report from a retrospective point -of -view the baseline characteristics of the study subjects who experienced AF during the follow-up. In this part of the study, subjects with missing ultrasonography data were also enrolled. Thereby, the total number of study subjects whose AF events were available was 969. It is worth mentioning that in the total OPERA cohort (n = 1,045 subjects) there was only one subject who was diagnosed with AF at baseline.

The study was approved by the Ethics Committee of the Medical Department of the University of Oulu.

### Determination of hepatic steatosis

The determination of hepatic steatosis was based on liver-kidney contrast assessed with ultrasonography [25] by one trained radiologist with 10 years’ experience of abdominal ultrasound examinations. Ultrasonography has good sensitivity and specificity to detect moderate to severe fat accumulation in the liver compared to liver biopsy, which is the golden standard for the diagnosis of NAFLD [26–28], but the sensitivity declines when the amount of fat in the liver is <33% [26]. Of the 1,045 OPERA study subjects this data was available on 1,028 subjects. The severity of hepatic steatosis was based on the brightness of the liver and it was classified into three groups ranging from 0 to 2 (0 = normal bright, indicating a non-fatty liver, 1 = medium bright, a moderate lipid content and 2 = clearly bright, a severe lipid content and fatty liver). In this article, we compared subjects with normal brightness of liver (group 0) with those with fatty liver (= combined groups 1 and 2).

### Follow-up

During the follow-up, diagnosis of AF (including atrial flutter) was made if this event was listed as ICD-10 code (I48) in the National Death Registry and/or hospital discharge registry (HILMO). Diagnosis of AF was based on standard 12-lead resting ECG. The follow-up time lasted until December 31, 2009, or the occurrence of the first event. The mean follow-up time was 16.3 years (median 17.6 years, range 0–19 years).

### Laboratory tests

All the laboratory test samples were obtained after an overnight fast. Blood insulin and glucose concentrations were analyzed at 0, 60, and 120 min after administration of 75 g glucose [29]. Insulin sensitivity was assessed using fasting plasma insulin concentrations and a quantitative insulin sensitivity check index (QUICKI) (QUICKI = 1/(log (fasting insulin)+log (fasting glucose))) [30]. Very-low-density lipoprotein (VLDL), high-density lipoprotein (HDL), low-density lipoprotein (LDL) and hs-CRP concentrations [29] as well as ALT and γ-GT levels were measured as described previously [24]. Plasma atrial natriuretic peptide (ANP) was determined by radioimmunoassay [31].

### Echocardiographic methods

A Hewlett-Packard ultrasound color system, Sonos 500 (Hewlett-Packard Company, Massachusetts, USA) was used for the echocardiographic examinations. All procedures were performed by one experienced cardiologist (Markku Ikäheimo), who was blinded to the other data and grouping of the study subjects. M-mode measurements were obtained under 2-D guidance according to the recommendations of the American Society of Echocardiography [32].

### Statistical methods

The statistical significances of differences in continuous and categorical variables between the patients with and without fatty liver and AF were assessed using the standard t-test and the chi-square test, respectively. Logarithmic transformations were used when variable distributions were not normal (quick index, ANP, hs-CRP, triglycerides, γ-GT, ALT, creatinine). The cumulative proportional probability of the development of AF requiring hospitalization is shown by the Kaplan-Myer curves. The Log Rank test was used to evaluate the statistical significance of the separation of the curves. The Cox hazards model was used to evaluate the univariate and multivariate significance of different factors in predicting new-onset AF requiring hospitalization. The data were analyzed using the IBM Statistics 22. A p-value <0.05 was considered to be statistically significant.

## Results

The baseline features of NAFLD and non-NAFLD patients are shown in detail in Table 1. In the OPERA cohort (n = 958) the total prevalence of NAFLD was 26.0% (n = 249). There were more male in the NAFLD group than in the non-NAFLD group. The mean age of patients in the NAFLD group and in the non-NAFLD group did not differ from each other. The presence of NAFLD increased the probability of belonging to the hypertensive study group. The patients with NAFLD were more obese, had more abdominal fat, smoked more, drank more alcohol, had higher systolic and diastolic blood pressure, higher values of γ-GT, ALT, hs-CRP and fasting glucose than those without NAFLD. Diabetes was also considerably more prevalent in subjects with NAFLD than in those without NAFLD, and QUICK index was lower in patients with NAFLD compared to the non-NAFLD subjects. Moreover, lipid status differed between the groups: in subjects with NAFLD total cholesterol and triglycerides were higher whereas HDL-cholesterol was lower. ANP was lower in the NAFLD group.

**Table 1:** Baseline characteristics of the study subjects (n = 958) according to the presence or absence of NAFLD.

	Non-NAFLD (n = 709)	NAFLD (n = 249)	p-value
Age (years)	51 ± 6	52 ± 6	0.085
Sex (female), n (%)	403 (57%)	105 (42%)	<0.001
Study group (hypertensives) (%)	297 (42%)	175 (70%)	<0.001
Diabetics n (%)	28 (4%)	69 (28%)	<0.001
Hypertension n (%)	312 (44%)	180 (72%)	<0.001
Coronary artery disease	50 (7%)	29 (12%)	0.034
Systolic blood pressure (mmHg)	145 ± 22	153 ± 20	<0.001
Diastolic blood pressure (mmHg)	87 ± 12	92 ± 10	<0.001
Body mass index (kg/m^2^)	26.4 ± 4	31.1 ± 5	<0.001
Waist circumference (cm)	86 ± 12	100 ± 12	<0.001
Fasting glucose (mmol/l)	4.4 ± 0.7	5.6 ± 2.4	<0.001
Quick index (l/mmol)	0.64 ± 0.11	0.52 ± 0.08	<0.001
ANP (pmol/l)	288 ± 172	264 ± 156	0.049
Cholesterol (mmol)	5.6 ± 1.0	5.8 ± 1.1	0.022
HDL-cholesterol (mmol/l)	1.4 ± 0.4	1.2 ± 0.3	<0.001
LDL-cholesterol (mmol/l)	3.5 ± 0.9	3.6 ± 1.0	0.116
hs-CRP (mg/l)	3.1 ± 6.7	5.3 ± 8.7	<0.001
Triglycerides (mmol/l)	1.4 ± 0.8	2.1 ± 1.2	<0.001
γ-GT (U/l)	34 ± 30	65 ± 87	<0.001
ALT (U/l)	26 ± 15	45 ± 26	<0.001
Creatinine (μmol/l)	82 ± 37	83 ± 15	0.666
Alcohol (g/week)	37 ± 46	55 ± 58	<0.001
Smoking (pack years)	8 ± 13	11 ± 14	0.002
Fractional shortening (%)	35 ± 6	35 ± 6	0.603
Left ventricular mass index (g/m^2^)	128±37	138 ± 39	0.001
Left atrial diameter (mm)	38 ± 5	41 ± 5	<0.001
β-blockers, n (%)	158 (22%)	103 (41%)	<0.001
Calcium blockers, n (%)	68 (10%)	47 (19%)	<0.001
ACE-inhibitors, n (%)	119 (17%)	58 (23%)	0.023
Diuretic drugs, n (%)	88 (12%) 4%	63 (25%)	<0.001
Digitalis, n (%)	9 (1%)	14 (6%)	<0.001
Lipid lowering drugs, n (%)	19 (3%)	10 (4%)	0.290
Aspirin, n (%)	38 (5%)	15 (6%)	0.693

The values are means ± SD, absolute numbers with percentages or percentages alone. The medication data is expressed as number of subjects and percentages. Differences were tested by the ANOVA test for continuous variables and Pearson Chi-Squared test for categorical variables. ANP, atrial natriuretic peptide; hs-CRP, high-sensitive C-reactive protein; γ-GT, gamma-glutamyl transpeptidase; ALT, alanine aminotransferase; ACE, angiotensin converting enzyme.

As regards echocardiographic measurements, patients with NAFLD had a greater diameter of the left atrium and left ventricular mass index than those without this condition.

CAD was more prevalent in the subjects with NAFLD than in non-NAFLD subjects. Subjects with NAFLD used more often digitalis, diuretics, beta blocking agents, calcium channel blockers and inhibitors of angiotensin converting enzyme (ACE-inhibitors) compared to the subjects in the non-NAFLD group.

Several baseline characteristics differed in subjects who experienced atrial fibrillation in the follow-up compared to those who did not. This is shown in Table 2. There were more men in the AF group than in the non-AF group. Furthermore, the subjects with AF were older, more obese, had more abdominal fat and higher systolic blood pressure, γ-GT, ALT, creatinine and ANP values. In addition, QUICK index and HDL-cholesterol level were lower with the subjects with AF. In addition, CAD and NAFLD were more prevalent in the AF group compared to the non-AF group. When the different types of medicines were analyzed, statistically significant differences were observed in the use of beta blockers, digitalis and ASA. Furthermore, subjects belonging to the NAFLD group had a larger left ventricular mass index and left atrial diameter compared to those without atrial fibrillation. There was no statistical difference in the distribution of the original OPERA study group (hypertension or control group), hypertension and diabetes status in the AF and non-AF groups.

**Table 2:** Baseline characteristics in the subjects (n = 969) with and without atrial fibrillation (AF) in the follow-up.

	No AF (n = 875)	AF (n = 94)	p-value
Age (years)	51 ± 6	54 ± 5	<0.001
Sex (female) n (%)	473 (54%)	39 (41%)	0.020
Study group (hypertensives) n (%)	426 (49%)	52 (55%)	0.222
Fatty liver in ultrasound n (%)*	212 (25%)	37 (40%)	0.001
Diabetics n (%)	86 (10%)	12 (13%)	0.369
Hypertension n (%)	442 (51%)	56 (60%)	0.095
Coronary artery disease (%)	63 (7%)	17 (18%)	<0.001
Mean systolic blood pressure (mmHg)	147 ± 21	154 ± 23	0.001
Mean diastolic blood pressure (mmHg)	89 ±12	90 ± 14	0.326
Body mass index (kg/m^2^)	27.5 ± 5	29.0 ± 5	0.003
Waist circumference (cm)	90 ± 13	95 ± 13	<0.001
Fasting glucose (mmol/l)	4.7 ± 1.5	4.8 ± 1.3	0.486
Quick index (l/mmol)	0.61 ± 0.12	0.58 ± 0.11	0.035
ANP (pmol/l)	274 ± 152	353 ± 266	<0.001
Total cholesterol (mmol/l)	5.7 ± 1.1	5.7 ± 1.0	0.546
HDL-cholesterol (mmol/l)	1.4 ± 0.4	1.3 ± 0.4	0.037
LDL-cholesterol (mmol/l)	3.5 ± 1.0	3.6 ± 0.9	0.410
Triglycerides (mmol/l)	1.5 ± 1.0	1.7 ± 1.0	0.086
hs-CRP (mg/l)	3.7 ± 7.5	3.7 ± 5.9	0.006
γ-GT (U/l)	41 ± 58	63 ± 94	0.001
ALT (U/l)	31 ± 21	36 ± 21	0.023
Creatinine (μmol/l)	81 ± 15	91 ± 94	0.006
Alcohol (g/week)	41 ± 49	46 ± 58	0.349
Smoking (pack years)	9 ± 13	11 ± 15	0.109
Fractional shortening (%)	35 ± 6	34 ± 6	0.084
Left ventricular mass index (g/m^2^)	128 ± 36	150 ± 42	<0.001
Left atrial diameter (mm)	39 ± 5	41 ± 5	0.001
β-blockers n (%)	226 (26%)	37 (39%)	0.005
Calcium blockers n (%)	104 (12%)	12 (13%)	0.803
ACE-inhibitors n (%)	158 (18%)	23 (24%)	0.130
Diuretic drugs n (%)	137 (16%)	17 (18%)	0.541
Digitalis n (%)	12 (1%)	11 (12%)	<0.001
Lipid lowering drug n (%)	26 (3%)	3 (3%)	0.905
Aspirin n (%)	44 (5%)	10 (11%)	0.024

The values are means ± SD, absolute numbers with percentages or percentages alone. The medication data is expressed as number of subjects and percentages. Differences were tested by the ANOVA test for continuous variables and Pearson Chi-Squared test for categorical variables. ANP, atrial natriuretic peptide; hs-CRP, high-sensitive C-reactive protein; γ-GT, gamma-glutamyl transpeptidase; ALT, alanine aminotransferase; ACE, angiotensin converting enzyme.

* Ultrasonography data available on 958 study subjects

In multivariate Cox regression analysis of the NAFLD group, see Table 3, relevant covariates (not medications and plasma lipids) were chosen as potential confounding factors on the basis of their significance in univariate analyses. In Model 1 the association between NAFLD and AF remained statistically significant after adjusting for age and sex. Further adjustment for diabetes and study group status did not remarkably change this association, as seen in Model 2. In Model 3 the following additional factors were included in the model: BMI, waist circumference, alcohol consumption, smoking (according to pack-years), serum ALT concentration, systolic blood pressure, quick index, LVMI (Left Ventricular Mass Index), left atrial diameter, ANP, CAD and hs-CRP. In this model, NAFLD (p = 0.041), older age (p = 0.009), higher LVMI (p = 0.016) and ANP (p = 0.004) were independently associated with AF.

**Table 3:** Association between NAFLD (n = 958) and risk of AF during follow-up.

	Cox regression model			
Predictor	Unadjusted model	Adjusted model 1	Adjusted model 2	Adjusted model 3
Fatty liver (yes vs. no)	**1.96 (1.29–2.97)**	**1.79 (1.18–2.71)**	**1.73 (1.09–2.73)**	**1.88 (1.03–3.45)**
Age (years)		**1.09 (1.05–1.13)**	**1.09 (1.05–1.13)**	**1.06 (1.01–1.11)**
Sex (male vs female)		**1.63 (1.07–2.49)**	**1.63 (1.07–2.49)**	0.78 (0.32–1.90)
Study group (hypertensive vs. control)			1.12 (0.73–1.71)	0.70 (0.40–1.23)
Diabetes status (yes vs no)			1.00 (0.53–1.91)	1.01 (0.48–2.13)
BMI (kg/m^2^)				0.91 (0.80–1.03)
Waist (cm)				1.03 (0.98–1.08)
Alcohol consumption (grams/week)				1.00 (0.99–1.00)
Smoking (pack years)				1.00 (0.98–1.02)
Serum ALT (U/l)				1.00 (0.99–1.01)
Systolic Blood Pressure (mmHg)				1.01 (1.00–1.02)
Quick Index				0.93 (0.06–14.90)
CAD, (yes vs no)				1.70 (0.86–3.39)
ANP (pmol/l)				**1.002 (1.000–1.003)**
LVMI (g/m^2^)				**1.01 (1.00–1.02)**
Left atrial diameter (mm)				1.03 (0.97–1.09)
hs-CRP (mg/l)				1.00 (1.00–1.00)

Values are expressed as ORs (95% CIs) as assessed by multivariate Cox regression analyses. Independent predictors of AF are highlighted in bold type. BMI, body mass index; ALT, alanine transferase; CAD, coronary artery disease; ANP, atrial natriuretic peptide; LVMI, left ventricular mass index; hs-CRP, high-sensitive C-reactive protein.

**Fig 1:**
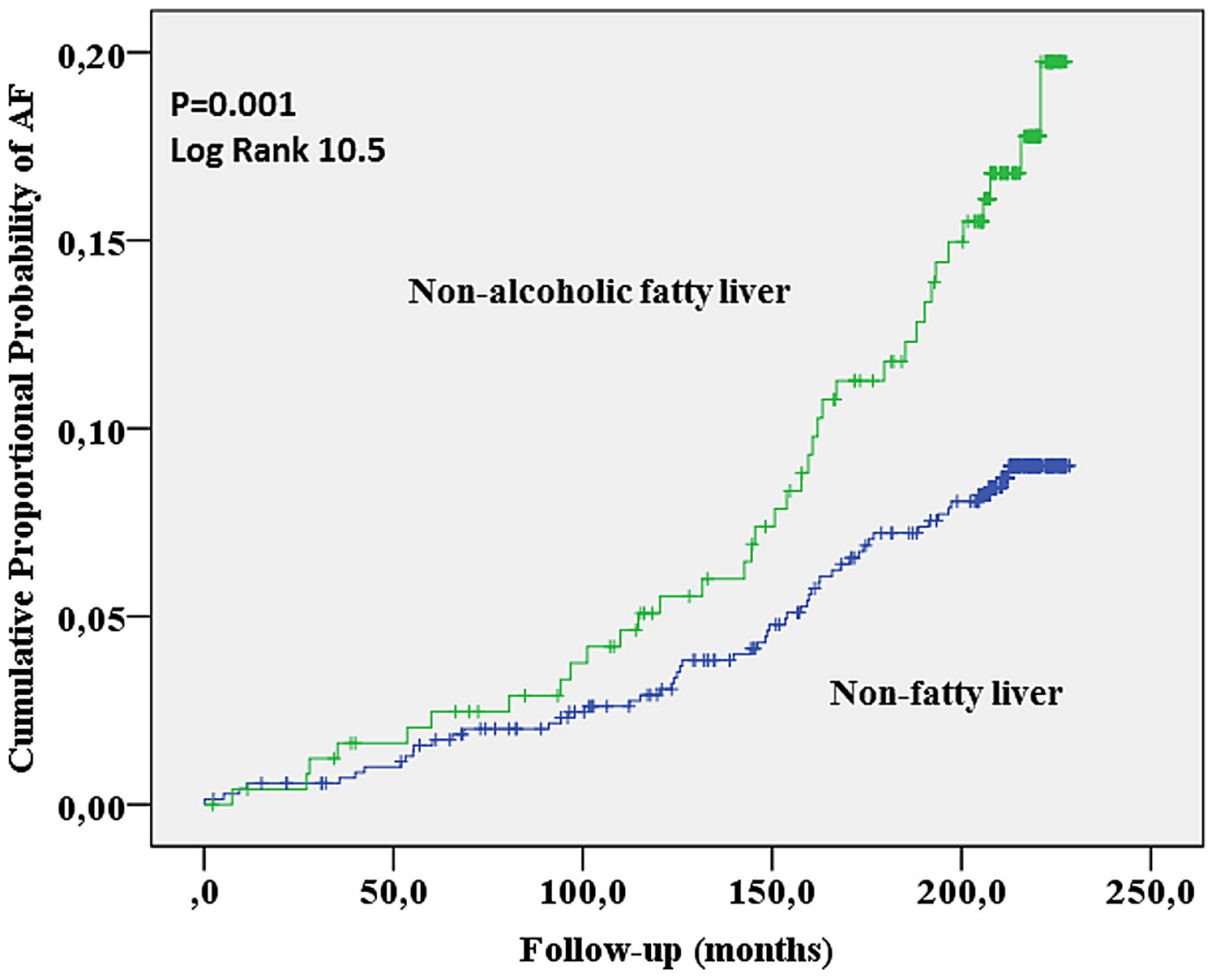
The cumulative proportional probability of AF in the NAFLD group.

Subjects in the NAFLD group had greater probability of developing atrial fibrillation during the follow-up time in comparison to the subjects in the non-NAFLD group (Hazard ratio (HR) 1.96 (95%CI) 1.29–2.97). After the follow-up time, 14.9% of subjects (n = 37) with NAFLD at baseline had been diagnosed with atrial fibrillation. Contrary to that, 7.9% of subjects (n = 56) without NAFLD at the baseline experienced atrial fibrillation (p = 0.001) during the follow-up time. Fig 1 shows the detailed cumulative proportional probability of AF in the NAFLD group.

## Discussion

Our main finding was that NAFLD was an independent risk factor of atrial fibrillation in this OPERA study of nearly 1,000 subjects with a long follow-up time. Whether this association is a causative one or whether these two entities just share common pathophysiologic mechanisms is unclear. Considering that NAFLD is a known risk factor for a broad spectrum of cardiovascular diseases, it is plausible to assume that there is a cause-and-effect link.

Our findings agree with the study by Targher et al. [20, 21]. These authors demonstrated in two different studies that NAFLD is associated with an increased prevalence and incidence of AF in patients with type II diabetes [20, 21]. The first [20] of these studies was a prospective 10-year follow-up study which pointed to an increased risk of incident AF in patients with NAFLD compared to the persons without NAFLD. This association seemed to be independent of the other risk factors (age, sex, hypertension, left ventricular hypertrophy and PR interval). The basis of this study was similar to ours (a follow-up study, NAFLD diagnosed by ultrasonography). However, there are some differences from our study as the follow-up time was shorter and the number of patients smaller in the study by Targher et al. No echocardiographic data were available, either; LV hypertrophy, for instance, was based on ECG Voltage criteria. Furthermore, the patients were older, and, due to all having type 2 diabetes, more obese. It is important to point out that in this study all patients had type 2 diabetes whereas in the OPERA study the diabetes status did not affect the inclusion and did not affect our results either. The latter study by Targher et al. [21] was cross-sectional and the initial setting was thus different from ours. However, the study showed that in hospitalized patients with type 2 diabetes NAFLD was associated with an increased prevalence of persistent or permanent AF. Once again, none of the adjustments for the other risk factors of AF (age, sex, systolic blood pressure, HbA1C%, estimated GFR, total cholesterol, ECG-based left ventricular hypertrophy, heart failure, COPD, valvular heart disease or hyperthyreoidism) attenuated this association [21]. There are also two studies showing that circulating levels of liver enzymes correlate with incidence of AF [22, 23]. The study by Alonso et al. [22] showed that γ-GT, a possible marker of NAFLD, was linearly associated with the risk of AF. Additionally, it is worth noting that the linear association between γ-GT and risk of AF was not altered after restricting the analysis to never-drinkers. Moreover, there is a study, based on the Framingham Heart Study, which had over 3,700 patients who were followed up to 10 years, showing that both transaminases (ALT and AST) were significantly associated with greater risk of incident AF [23]. The association was independent of standard AF risk factors and alcohol consumption. All subjects were also free from clinical heart failure. This association remained even after adjustment for clinically relevant interim heart failure during the follow-up. As noted earlier, in our study γ-GT and ALT were predictors of AF as well. All these studies suggest NAFLD to be an independent risk factor for AF in both diabetic and non-diabetic population.

The mechanisms that link NAFLD to atrial fibrillation are not completely understood. It is clear that there are common risk factors and co-morbidities.

First, NAFLD causes systemic inflammation [33, 34] by several mechanisms. The energy surplus leads to fat accumulation in the hepatocytes, which, in turn, promotes oxidative stress and secretes inflammatory factors. An increasing state of inflammation locally in the liver leads to formation of non-alcoholic steatohepatitis (NASH) [35–37] and may predispose to systemic inflammation. Inflammation is a potent trigger of AF through various processes [38–41] and, vice versa, AF seems to produce and sustain a pro-inflammatory environment [40]. Therefore, the relation between AF and systemic inflammation is thought to be bidirectional.

Second, NAFLD seems to be an independent risk factor of autonomic dysfunction in the Chinese population [42], and there are also other reports of this association [43, 44]. The variation of symphatovagal activation seems to be profibrillatory in the initiation and maintenance of atrial fibrillation [45]. According to some reports, autonomic dysfunction is a risk factor of atrial fibrillation [45–47]. These associations between NAFLD and autonomic dysfunction and, moreover, between autonomic dysfunction and AF, may provide a causal link between NAFLD and AF.

Third, NAFLD has been shown to be associated with cardiac diastolic dysfunction [48–50], which, in turn, is reported to provoke atrial fibrillation through various mechanisms [51, 52]. There are also reports that type 2 diabetes, but not NAFLD, causes diastolic dysfunction [53] and that NAFLD is associated with diastolic dysfunction if type 2 diabetes also exists [54, 55]. In our study, patients suffering from atrial fibrillation during the follow-up had a higher left ventricular mass index at baseline, and in multivariate model baseline left ventricular mass was a significant predictor of atrial fibrillation in the NAFLD group. Unfortunately, left ventricular diastolic dysfunction was not measured at baseline in our cohort.

There are a few limitations in our study.

First, one may argue as to the validity of the diagnosis of AF based on the National Death Registry and hospital discharge registry due to the fact that AF is often asymptomatic and/or paroxysmal, and thereby it is impossible to know all the AF cases. However, adequate validity of this method has been shown in epidemiological studies [56, 57].

Second, owing to the study design (OPERA study) [24], our study population may have had higher prevalence of hypertension than the general population as half of the study population had a diagnosis of hypertension. A baseline population free from cardiovascular disease would have been the best option to study the risk factors of the incidence of AF. However, it is worth noting that the distribution of NAFLD, but not the distribution of AF, differed statistically significantly according to the original OPERA study group (subjects with hypertension medication versus subjects without hypertension medication). In addition, the study group was included in the adjustments when the association between NAFLD and AF was analyzed.

Third, the indication for the use of digitalis was not documented, which may raise the question of whether there were subjects with unknown AF. The total number of digitalis users without known AF diagnosis was 12. Presumably these subjects had chronic heart failure, because we re-checked the original OPERA cohort data (n = 1,045) and there was only one AF diagnosis at baseline.

Fourth, due to the original study design of the OPERA study [24], we lack the data on secondary causes of hepatic steatosis, such as viral causes of liver diseases. The prevalence of hepatitis viruses in Finland is, however, so low [58] that it is unlikely that missing data on viral hepatitis would have changed our final results.

In conclusion, the present study provides strong epidemiological evidence that NAFLD is an independent risk factor for atrial fibrillation. Additional studies are needed to demonstrate the pathophysiological mechanisms underlying this association.

## Abbreviations

(NAFLD): Non-alcoholic fatty liver disease
(AF): Atrial Fibrillation
(CAD): Coronary Artery Disease

## References

[1] Targher GT, Day CP, Bonora E. Risk of Cardiovascular Disease in Patients with Nonalcoholic fatty Liver Disease. N Engl J Med. 2010;363: 1341–50. 10.1056/NEJMra0912063 20879883

[2] Neuschwander-Tetri BA, Caldwell SH. Nonalcoholic steatohepatitis: summary of an AASLD Single Topic Conference. Hepatology. 2003;37: 1202–1219. 12717402 10.1053/jhep.2003.50193

[3] Baumeister SE, Volzke H, Marchall P, John U, Schmidt CO, Flessa S, et al Impact of fatty liver disease on health care utilization and costs in a general population: A 5-year observation. Gastroenterology. 2008;134: 85–94. 18005961 10.1053/j.gastro.2007.10.024

[4] Liu H, Lu H-Y. Nonalcoholic fatty liver disease and cardiovascular disease. World J Gastroenterol. 2014 7 14;20(26): 8407–8415. 10.3748/wjg.v20.i26.8407 25024598 PMC4093693

[5] Bhatia LS, Curzen NP, Calder PC, Byrne CD. Non-alcoholic fatty liver disease: a new and important cardiovascular risk factor. Eur Heart Journal. 2012 5;33(10): 1190–1200. 10.1093/eurheartj/ehr453 22408036

[6] Ballestri S, Lonardo A, Bonapace S, Byrne CD, Loria P, Targher G. Risk of cardiovascular cardiac and arrhythmic complications in patients with non-alcoholic fatty liver disease. World J Gastroenterol. 2014;20: 1724–1745. 10.3748/wjg.v20.i7.1724 24587651 PMC3930972

[7] Lonardo A, Ballestri S, Marchesini G, Angulo P, Loria P. Nonalcoholic fatty liver disease: A precursor of the metabolic syndrome. Dig Liver Dis. 2015;47: 181–90. 10.1016/j.dld.2014.09.020 25739820

[8] Yki-Järvinen H. Non-alcoholic fatty liver disease as a cause and consequence of metabolic syndrome. Lancet Diabetes Endocrinol. 2014 11;2(11): 901–10. 10.1016/S2213-8587(14)70032-4 24731669

[9] Nascimbeni F, Pais R, Bellentani S, Day CP, Ratziu V, Loria P, et al From NAFLD in clinical practice to answers from guidelines. J Hepatol. 2013 10;59(4): 859–71. 10.1016/j.jhep.2013.05.044 23751754

[10] Lip GY, Tse HF, Lane DA. Atrial fibrillation. Lancet. 2012;379: 648–661. 10.1016/S0140-6736(11)61514-6 22166900

[11] Miyasaka Y, Barnes ME, Gersh BJ, Cha SS, Bailey KR, Abhayaratna WP. Secular trends in incidence of atrial fibrillation in Olmsted Country, Minnesota, 1980 to 2000, and implications on the projections for future prevalence. Circulation. 2006;114(2): 119–125. 16818816 10.1161/CIRCULATIONAHA.105.595140

[12] Lloyd-Jones DM, Wang TJ, Leip EP, Larson MG, Levy D, Vasan RS, et al Lifetime risk for development of atrial fibrillation: The Framingham Heart Study. Circulation. 2004;110: 1042–1046. 15313941 10.1161/01.CIR.0000140263.20897.42

[13] Nichols GA, Reinier K, Chugh SS. Independent contribution of diabetes to increased prevalence and incidence of atrial fibrillation. Diabetes Care. 2009;32: 1851–6. 10.2337/dc09-0939 19794003 PMC2752931

[14] Benjamin EJ, Levy D, Vaziri SM, D’Agostino RB, Belanger AJ, Wolf PA. Independent risk factors for atrial fibrillation in a population-based cohort: the Framingham Heart Study. JAMA, J. Am. Med. Assoc. 1994;271: 840–844. 8114238

[15] Yamashita T. Recent Mortality and Morbidity Rates of Japanese Atrial Fibrillation Patients–Racial Differences and Risk Stratification. Circ J. 2013;77(4): 864–8. 23449371 10.1253/circj.cj-13-0002

[16] Chugh SS, Havmoeller R, Narayanan K, Singh D, Rienstra M, Benjamin EJ, et al Worldwide epidemiology of atrial fibrillation: a Global Burden of Disease 2010 Study. Circulation. 2014 2 25;129(8): 837–47. 10.1161/CIRCULATIONAHA.113.005119 24345399 PMC4151302

[17] Suzuki S Yamashita T, Otsuka T, Sagara K, Uejima T, Yajima J, et al Recent mortality of Japanese patients with atrial fibrillation in an urban city of Tokyo. J Cardiol. 2011 9;58(2): 116–23. 10.1016/j.jjcc.2011.06.006 21820280

[18] Ohsawa M, Okayama A, Okamura T, Itai K, Nakamura M, Tanno K, et al Mortality risk attributable to atrial fibrillation in middle-aged and elderly people in the Japanese general population; Nineteen-year follow-up in NIPPON DATA80. Circ J. 2007;71: 814–9. 17526974 10.1253/circj.71.814

[19] Nieuwlaat R, Prins MH, Le Heuzey J-Y, Vardas PE, Aliot E, Santini M, et al Prognosis, disease progression, and treatment of atrial fibrillation patients during 1 year: follow-up of the Euro Heart Survey on Atrial Fibrillation. Eur Heart J. 2008 5;29(9): 1181–9. 10.1093/eurheartj/ehn139 18397874

[20] Targher G, Valbusa F, Bonapace S, Bertolini L, Zenari L, Rodella S, et al Non-alcoholic fatty liver disease is associated with an increased incidence of atrial fibrillation in patients with type 2 diabetes. PLoS One. 2013;8(2):e57183 10.1371/journal.pone.0057183 Epub 2013 Feb 22. 23451184 PMC3579814

[21] Targher G, Mantovani A, Pichiri I, Rigolon R, Dauriz M, Zoppini G, et al Non-alcoholic fatty liver disease is associated with an increased prevalence of atrial fibrillation in hospitalized patients with Type 2 diabetes. Clinical Science. 2013;125: 301–309. 10.1042/CS20130036 23596966

[22] Alonso A, Misialek JR, Amiin MA, Hoogeveen RC, Chen LY, Agarwal SK, et al Circulating levels of liver enzymes and incidence of atrial fibrillation: the Athersclerosis Risk in Communities cohort. Heart. 2014;100: 1511–1516. 10.1136/heartjnl-2014-305756 24924619 PMC4225783

[23] Sinner MF, Wang N, Fox CS, Fontes JD, Rienstra M, Magnani JW, et al Relation of Circulating Liver Transaminase Concentrations to Risk of New-onset Atrial Fibrillation. Am J Cardiol. 2013 1 15;111(2): 219–224. 10.1016/j.amjcard.2012.09.021 23127690 PMC3538882

[24] Rantala AO, Kauma H, Lilja M, Savolainen MJ, Reunanen A, Kesäniemi YA. Prevalence of the metabolic syndrome in drug-treated hypertensive patients and control subjects. Journal of Internal Medicine. 1999;245: 163–174. 10081519 10.1046/j.1365-2796.1999.00429.x

[25] Ballestri S, Romagnoli D, Nascimbeni F, Francica G, Lonardo A. Role of ultrasound in the diagnosis and treatment of nonalcoholic fatty liver disease and its complications. Expert Rev Gastroenterol Hepatol. 2015 5;9(5): 603–27. 10.1586/17474124.2015.1007955 25694178

[26] Mehta SR, Thomas EL, Bell JD, Johnston DG, Taylor-Robinson SD. Non-invasive means of measuring hepatic fat content. World J Gastroenterol. 2008;14: 3476–3483. 18567074 10.3748/wjg.14.3476 PMC2716608

[27] Nalbantoglu I, Brunt E. Role of liver biopsy in nonalcoholic fatty liver disease. World J Gastroenterol. 2014 7 21;20(27): 9026–9037. 10.3748/wjg.v20.i27.9026 25083076 PMC4112884

[28] Hernaez R, Lazo M, Bonekamp S, Kamel I, Brancati FL, Guallar E, et al Diagnostic Accuracy and Reliability of Ultrasonography for the Detection of Fatty Liver: A Meta-Analysis. Hepatology. 2011 9 2;54(3): 1082–1090. 10.1002/hep.24452 21618575 PMC4197002

[29] Pisto P, Ukkola O, Santaniemi M, Kesaniemi YA. Plasma adiponectin—an independent indicator of liver fat accumulation. Metabolism. 2011;11; 60(11): 1515–20. 10.1016/j.metabol.2011.03.009 21565369

[30] Pisto P, Santaniemi M, Bloigu R, Ukkola O, Kesäniemi YA. Fatty liver predicts the risk for cardiovascular events in middle-aged population: a population-based cohort study. BMJ Open. 2014 3 20;4(3):e004973 10.1136/bmjopen-2014-004973 24650811 PMC3963104

[31] Karjalainen AH, Ruskoaho H, Vuolteenaho O, Heikkinen JE, Bäckström AC, Savolainen MJ, Kesäniemi YA. Effects of estrogen replacement therapy on natriuretic peptides and blood pressure. Maturitas. 2004 3 15;47(3): 201–8. 15036490 10.1016/S0378-5122(03)00279-2

[32] Lang RM, Badano LP, Mor-Avi V, Afilalo J, Armstrong A, Ernande L, et al Recommendations for cardiac chamber quantification by echocardiography in adults: an update from the American society of echocardiography and the European association of cardiovascular imaging. Eur Heart J Cardiovasc Imaging. 2015;16: 233–71. 10.1093/ehjci/jev014 25712077

[33] Ndumele CE, Nasir K, Conceiçao RD, Carvalho JA, Blumenthal RS, Santos RD. Hepatic steatosis, obesity, and the metabolic syndrome are independently and additively associated with increased systemic inflammation. Arterioscler Thromb Vasc Biol. 2011 8;31(8): 1927–32. 10.1161/ATVBAHA.111.228262 21546603 PMC3148106

[34] Targher G, Bertolini L, Rodella S, Lippi G, Franchini M, Zoppini G, et al NASH predicts plasma inflammatory biomarkers independently of visceral fat in men. Obesity (Silver Spring). 2008 6;16(6): 1394–9. 18369343 10.1038/oby.2008.64

[35] Verdelho Machado M, Cortez-Pinto H. Non-alcoholic fatty liver disease: What the clinician needs to know. World J Gastroenterol. 2014 9 28;20(36): 12956–12980. 10.3748/wjg.v20.i36.12956 25278691 PMC4177476

[36] Furukawa S, Fujita T, Shimabukoro M, Iwaki M, Yamada Y, Nakajima Y, et al Increased oxidative stress in obesity and its impact on metabolic syndrome. J Clin Invest. 2004;114: 1752–1761. 15599400 10.1172/JCI21625 PMC535065

[37] Tateya S, Kim F, Tamori Y. Recent advances in obesity-induced inflammation and insulin resistance. Front Endocrinol (Lausanne) 2013 8 8;4:93 10.3389/fendo.2013.00093 eCollection 2013. 23964268 PMC3737462

[38] Chung MK, Martin DO, Sprecher D, Wazni O, Kanderian A, Carnes CA, et al C-reactive protein elevation in patients with atrial arrhythmias: inflammatory mechanisms and persistence of atrial fibrillation. Circulation. 2001 12 11;104(24): 2886–91. 11739301 10.1161/hc4901.101760

[39] Aviles RJ, Martin DO, Apperson-Hansen C, Houghtaling PL, Rautaharju P, Kronmal RA, et al Inflammation as a risk factor for atrial fibrillation. Circulation. 2003 12 16;108(24): 3006–10. Epub 2003 Nov 17. 14623805 10.1161/01.CIR.0000103131.70301.4F

[40] Guo Y, Lip GY, Apostolakis S. Inflammation in atrial fibrillation. J Am Coll Cardiol. 2012 12 4;60(22): 2263–70. 10.1016/j.jacc.2012.04.063 23194937

[41] Harada M, Van Wagoner DR, Nattel S. Role of Inflammation in Atrial Fibrillation Pathophysiology and Management. Circ J. 2015;79: 495–502. 10.1253/circj.CJ-15-0138 25746525 PMC4457364

[42] Sun W, Zhang D, Sun J, Xu B, Sun K, Wang T, et al Association between non-alcoholic fatty liver disease and autonomic dysfunction in a Chinese population. QJM. 2015 1 21. pii: hcv006. [Epub ahead of print]. 10.1093/qjmed/hcv006 25614616

[43] Newton J, Pairman J, Wilton K, Jones D, Day C. Fatigue and autonomic dysfunction in non-alcoholic fatty liver disease. Clin Auton Res. 2009;19: 319–326. 10.1007/s10286-009-0031-4 19768633

[44] Liu YC, Hung CS, Wu YW, Lee YC, Lin YH, Lin C, et al Influence of non-alcoholic fatty liver disease on autonomic changes evaluated by the time domain, frequency domain, and symbolic dynamics of heart rate variability. PLoS One. 2013 4 23;8(4):e61803 10.1371/journal.pone.0061803 Print 2013. 23626730 PMC3633992

[45] Shen MJ, Zipes DP. Role of autonomic Nervous System in Modulating Cardiac arrhythmias. Circ Res. 2014 3 14;114(6): 1004–21. 10.1161/CIRCRESAHA.113.302549 24625726

[46] Park HW, Shen MJ, Lin SF, Fishbein MC, Chen LS, Chen PS. Neural mechanisms of atrial fibrillation. Curr Opin Cardiol. 2012 1;27(1): 24–8. 10.1097/HCO.0b013e32834dc4e8 22139702 PMC3279730

[47] Perkiömäki J, Ukkola O, Kiviniemi A, Tulppo M, Ylitalo A, Kesäniemi YA, et al Heart rate variability findings as a predictor of atrial fibrillaton in middle-aged population. J Cardiovasc Electrophysiol. 2014 7;25(7): 719–24. 10.1111/jce.12402 24602026

[48] Granér M, Nyman K, Siren R, Pentikäinen MO, Lundbom J, Hakkarainen A, et al Ectopic fat depots and left ventricular function in nondiabetic men with nonalcoholic fatty liver disease. Circ Cardiovasc Imaging. 2014 12 30;8(1). pii: e001979. 10.1161/CIRCIMAGING.114.001979 Print 2015 Jan. 25550397

[49] Petta S, Argano C, Colomba D, Cammà C, Di Marco V, Cabibi D, et al Epicardial fat, cardiac geometry and cardiac function in patients with non-alcoholic fatty liver disease: Association with the severity of liver disease. J Hepatol. 2015 4;62(4): 928–33. 10.1016/j.jhep.2014.11.030 Epub 2014 Nov 28. 25445395

[50] Fotbolcu H, Yakar T, Duman D, Karaahmet T, Tigen K, Cevik C, et al Impairment of the left ventricular systolic and diastolic function in patients with non-alcoholic fatty liver disease. Cardiol J. 2010;17(5): 457–63. 20865675

[51] Tsang TS, Gersh BJ, Appleton CP, Tajik AJ, Barnes ME, Bailey KR, et al Left ventricular diastolic dysfunction as a predictor of the first diagnosed nonvalvular atrial fibrillation in 840elderly men and women. J Am Coll Cardiol. 2002 11 6;40(9): 1636–44. 12427417 10.1016/s0735-1097(02)02373-2

[52] Nagarakanti R, Ezekowitz M. Diastolic dysfunction and atrial fibrillation. J Interv Card Electrophysiol. 2008 8;22(2): 111–8. 10.1007/s10840-008-9203-8 Epub 2008 Feb 9. 18264747

[53] Cassidy S, Hallsworth K, Thoma C, MacGowan GA, Hollingsworth KG, Day CP, et al Cardiac structure and function are altered in type 2 diabetes and Non-alcoholic fatty liver disease and associate with glycemic control. Cardiovasc Diabetol. 2015 2 13;14:23 10.1186/s12933-015-0187-2 25849783 PMC4330943

[54] Bonapace S, Perseghin G, Molon G, Canali G, Bertolini L, Zoppini G, et al Nonalcoholic fatty liver disease is associated with left ventricular diastolic dysfunction in patients with type 2 diabetes. Diabetes Care. 2012 2;35(2): 389–95. 10.2337/dc11-1820 Epub 2011 Dec 30. 22210573 PMC3263884

[55] Mantovani A, Pernigo M, Bergamini C, Bonapace S, Lipari P, Pichiri I, et al Nonalcoholic Fatty Liver Disease Is Independently Associated with Early Left Ventricular Diastolic Dysfunction in Patients with Type 2 Diabetes. Plos One. 2015 8 7;10(8):e0135329 10.1371/journal.pone.0135329 eCollection 2015. 26252899 PMC4529087

[56] Alonso A, Agarwal SK, Soliman EZ, Ambrose M, Chamberlain AM, Prineas RJ, et al Incidence of atrial fibrillation in whites and African-Americans: the Atherosclerosis Risk in Communities (ARIC) study. Am Heart J. 2009;158: 111–17. 10.1016/j.ahj.2009.05.010 19540400 PMC2720573

[57] Jensen PN, Johnson K, Floyd J, Heckbert SR, Carnaham R, Dublin S. Identifying atrial fibrillation from electronic medical data: a systematic review. Pharmacoepidemiol Drug Saf. 2012;21(Suppl 1): 141–7. 22262600 10.1002/pds.2317 PMC3674852

[58] Färkkilä M. Drug therapy of hepatitis B and C. Duodecim. 2003;119(6): 519–29. (Article in Finnish, abstract in English.) 12708339

